# Stronger T Cell Immunogenicity of Ovalbumin Expressed Intracellularly in Gram-Negative than in Gram-Positive Bacteria

**DOI:** 10.1371/journal.pone.0065124

**Published:** 2013-05-31

**Authors:** Anna Martner, Sofia Östman, Samuel Lundin, Carola Rask, Viktor Björnsson, Esbjörn Telemo, L. Vincent Collins, Lars Axelsson, Agnes E. Wold

**Affiliations:** 1 Department of Infectious Diseases, University of Gothenburg, Gothenburg, Sweden; 2 Department of Medical Microbiology and Immunology, Institute of Biomedicine, University of Gothenburg, Gothenburg, Sweden; 3 Department of Rheumatology and Inflammation Research, Institute of Medicine, University of Gothenburg, Gothenburg, Sweden; 4 Nofima – Norwegian Institute of Food, Fisheries and Aquaculture Research, Ås, Norway; INRS - Institut Armand Frappier, Canada

## Abstract

This study aimed to clarify whether Gram-positive (G+) and Gram-negative (G−) bacteria affect antigen-presenting cells differently and thereby influence the immunogenicity of proteins they express. Lactobacilli, lactococci and *Escherichia coli* strains were transformed with plasmids conferring intracellular ovalbumin (OVA) production. Murine splenic antigen presenting cells (APCs) were pulsed with washed and UV-inactivated OVA-producing bacteria, control bacteria, or soluble OVA. The ability of the APCs to activate OVA-specific DO11.10 CD4^+^ T cells was assessed by measurments of T cell proliferation and cytokine (IFN-γ, IL-13, IL-17, IL-10) production. OVA expressed within *E. coli* was strongly immunogenic, since 500 times higher concentrations of soluble OVA were needed to achieve a similar level of OVA-specific T cell proliferation. Furthermore, T cells responding to soluble OVA produced mainly IL-13, while T cells responding to *E. coli*-expressed OVA produced high levels of both IFN-γ and IL-13. Compared to *E. coli*, G+ lactobacilli and lactococci were poor inducers of OVA-specific T cell proliferation and cytokine production, despite efficient intracellular expression and production of OVA and despite being efficiently phagocytosed. These results demonstrate a pronounced difference in immunogenicity of intracellular antigens in G+ and G− bacteria and may be relevant for the use of bacterial carriers in vaccine development.

## Introduction

Antigen-presenting cells (APCs) represent a link between innate and adaptive immunity by taking up soluble or particulate antigen and presenting them to T cells. Due to their effects on antigen-presenting cells, microbial antigens are typically more immunogenic than soluble non-microbial antigens, e.g. simple food proteins [1]. Firstly, antigens in the particulate format, such as microbes, are generally taken up more readily than soluble antigens by APCs [2], [3]. Secondly, conserved microbial structures, “danger signals”, stimulate pattern recognition receptors, leading to activation of the antigen-presenting cell with ensuing production of T cell activating cytokines and expression of co-stimulatory molecules. These signals promote activation, proliferation and cytokine production of naïve T cells that recognize their cognate antigen on the APC, while an encounter of the same antigen in the absence of such co-stimulatory signals results in T cell anergy [4], [5].

Gram-positive (G+) bacteria have a thick and rigid cell wall consisting of up to 50 layers of peptidoglycan along with teichoic acid, lipoteichoic acids, lipoproteins and other constituents whereas Gram-negative (G−) bacteria have a very thin peptidoglycan layer and an outer membrane that contains LPS and lipoproteins. LPS, peptidoglycan, lipoproteins, and lipotechoic acids are recognized by pattern recognition receptors (PPRs), many of which are expressed on different subsets of APCs [6]. In monocytes, intact G+ bacteria induce higher levels of IL-12 and TNF production than do G− bacteria, while the latter induce higher levels of IL-10 and PGE_2_ [7], [8], [9], [10]. Conversely, human monocytes that have been differentiated *in vitro* into dendritic cells (DCs) produce similar levels of IL-12 and TNF in response to G+ and G− bacteria [11], [12], [13]. Furthermore, G− bacteria are more potent than G+ bacteria in promoting the up-regulation of co-stimulatory molecules on monocyte-derived DCs [12], [13]. Presentation of unrelated antigens by DC to T cells is known to be enhanced by bacterial-induced maturation, and, in particular G− bacteria, have been shown to promote Th1 polarisation [13], [14], [15].

Despite these recognized differences in reactions of APCs to G+ and G− bacteria, little is known regarding how this affects the ability of APCs to boost and modulate T cell responses to an antigen expressed within the bacterium. To address this question, we cloned a fragment of the gene encoding the model antigen ovalbumin (OVA) into plasmids enabling its production intracellularly in G+ (*Lactobacillus sakei*, *Lactobacillus plantarum,* and *Lactococcus lactis*) and G− (*Escherichia coli*) bacteria. OVA-transformed bacteria, or soluble OVA, were fed to DCs derived from mouse spleen followed by assessment of proliferation and cytokine production by transgenic OVA-specific CD4^+^ T cells.

## Materials and Methods

### Ethics Statement

The experiments were carried out according to the guidelines of the ‘Council of Europe Convention for the Protection of Vertebrate Animals used for Experimental and Other Scientific purposes’. The study was approved by the Regional Ethics Committee, University of Gothenburg (Permit Number: 408-2008).

### Bacterial Strains and Media

The bacterial strains used in this study are listed in Table 1. *E. coli* HB101 (Promega, Madison, WI) was used as the host strain for a plasmid that encodes full-length ovalbumin (OVA), whereas *Lactobacillus sakei* Lb790 [16], *Lactobacillus plantarum* NC8 [17], *Lactococcus lactis* MG1363 [18], and *E. coli* XL10 Gold (Stratagene) were used as hosts for vectors encoding an immunodominant OVA fragment (OVA_f_) or green fluorescent protein (GFP). *E. coli* bacteria were grown at 37°C on BHI agar or in BHI broth (Oxoid, Basingstoke, UK) with shaking. Lactobacilli were cultured on MRS agar or in MRS broth (Oxoid). Lactococci were grown at 30°C on M17 agar or in static M17 broth (Oxoid) supplemented with 0.5% glucose. When appropriate, antibiotics were added to the growth medium, *i.e.*, 200 µg/ml erythromycin, 15 µg/ml chloamphenicol or 100 µg/ml ampicillin for *E. coli*, and 10 µg/ml erythromycin for the lactobacilli and lactococci.

**Table 1:** Bacterial strains and plasmids.

Strain/plasmid	Relevant characteristics	Source/Reference
**Strains**		
*Escherichia coli* HB101	G− host strain	Promega
*Escherichia coli* XL10 Gold	G− host strain	Stratagene
*Lactobacillus sakei* Lb790	G+ host strain	[16]
*Lactobacillus plantarum* NC8	G+ host strain	[17]
*Lactococcus lactis* MG1363	G+ host strain	[18]
**Plasmids**		
pOMP21	L8UV5 *lac* operator constitutive expression vector with lacZ::OVA, Amp^R^	[28], [29]
pIAβ8	Broad range vector; ColE1_rep_ (for replication in *E. coli* strains) and pAMβ1_rep_(for for replication in G+ strains), lacZ; T1T2; Cm^R^; Amp^R^	[30]
pIAβ8A	pIAβ8 without lacZ	Present study
pIAβ8A-OVA	pIAβ8A with lacZ::OVA	Present study
pSIP401	*spp*-based expression vector; *sppKR* expression driven by *ermB* read-throughand cognate promoter; 256_rep_; Em^R^	[31]
pSIP411	pSIP401 with SH71_rep_ and P_sppQ_::*gusA*	[32]
pSIP411-OVA_f_	pSIP411 with P_sppQ_::OVA_f_	Present study
pSIP409	pSIP401 with P_sppQ_::*gusA*	[32]
pSIP409p9	pSIP409 with p9::*gusA*	[33]
pSIP409p9-OVA_f_	pSIP409 with p9::OVA_f_	Present study
pSIP409p9-GFP	pSIP409 with p9::GFP	Axelsson (unpublished)
pSIP411p9-OVA_f_	pSIP411 with p9::OVA_f_	Present study
pSIP411p9-GFP	pSIP411 with p9::GFP	Present study

Amp^R^, ampicillin resistance; Cm^R^, chloramphenicol resistance; OVA_f_, fragment of the *OVA* gene; GFP, green fluorescent protein.

### Construction of OVA-producing Bacteria

The plasmids used in the present study are listed in Table 1. Plasmid DNA was isolated using the QIAprep Miniprep kit (Qiagen, Hilden, Germany), and recombinant plasmids were constructed by cloning [19] using restriction enzymes and T4 DNA ligase (Promega and New England Biolabs) and appropriate primers (MWG-Biotech AG, Ebersberg, Germany). PCR analyses were performed with the Gene Amp PCR System 9700 (Perkin-Elmer Biosystem) and Expand High Fidelity PCR System Polymerase (Roche Diagnostics). Details regarding construction of the plasmids are found in Methods S1.

### *E. coli* Producing Full-length OVA Protein

Production of OVA by the recombinant *E. coli* was verified by Western blotting of sonicated overnight cultures (2 min, amplitude: 60, Vibra-Cell ultrasonic processor; Sonics & Materials Inc., Newtown, CT). The sonicates, soluble OVA (Sigma Chemical Co., St Louis, MO), and a molecular mass marker (LMW electrophoresis calibration kit; Phamacia Biotech, Uppsala, Sweden) were separated by SDS-PAGE, transferred to nylon membranes, blocked with 2% donkey serum, treated with avidin-biotin (Vector Laboratories, Burlingame, CA), and stained with goat-anti-OVA antibodies (1∶2,000; Cappel, Durham, NC), followed by biotinylated donkey anti-goat-IgG antibodies (1∶5,000; Jackson Laboratories, West Grove, PA), ABC/HRP (DAKO, Glostrup, Denmark), and 0.5 mg/ml of the substrate diaminbenzidine (DAB) (Sigma).

The levels of full-length OVA produced by *E. coli* bacteria were also quantified by ELISA. Costar plates (Invitrogen, San Diego, CA) were coated with rabbit anti-ovalbumin antibody (1∶500; Immunology Consultants Laboratory, Newberg, OR) and blocked with 5% BSA (Sigma). Sonicated bacteria or OVA standard (Sigma) were diluted and detected using anti-ovalbumin HRP (1∶60,000; Nordic BioSite, Täby, Sweden), followed by tetramethylbenzidene (TMB) substrate (Sigma). The reaction was stopped with 1 M H_2_SO_4_, and the optical density of the solution was measured spectrophotometrically at 450 nm (Molecular Devices Corporation, Sunnyvale, CA).

### Construction of Lactobacilli, Lactococci, and *E. coli* Producing a Synthetic OVA Fragment or GFP

A synthetic gene that encodes amino acids 319–386 of chicken ovalbumin (termed OVA fragment; OVA_f_), and which was adapted with respect to the codon usage of lactobacilli (Fig. S1), was generated. The gene was flanked by the restriction sites *Nco*I (start codon) and *Xho*I, and was inserted into the multiple cloning site of the pUC57 plasmid (GeneScripts Corp., Piscataway, NJ). Details regarding the construction of plasmids are found in Methods S1.

Production of the OVA fragment was semiquantitatively estimated by SDS-PAGE. Transformed bacteria were sonicated with glass beads (≤106 µm; Sigma) [20], and 350 ng of bacterial proteins, as determined using the NanoDrop system (NanoDrop, Wilmington, DE), were separated by SDS-PAGE and transferred onto PVDF membranes (Hybond P; Amersham Biosciences). Membranes were blocked with 5% BSA, and stained with rabbit α-ovalbumin antibody (1∶1,000, ICL, UK), followed by AP-conjugated anti-rabbit IgG (1∶7,500; Promega) and NBT/BCIP (Promega). His-tagged OVA_f_ (see Methods S1) was used as a molecular mass marker.

GFP expression was measured by of serial dilutions of lysates using the Typhoon 8600 Imager (GE Healthcare/Amersham Biosciences, Pittsburgh, PA, USA).

### Preparation of OVA-producing Bacteria and Soluble OVA for Use in DC-T cell Co-cultures

Recombinant bacteria were cultured overnight with appropriate antibiotics, diluted to an OD_600_ of ∼0.05 and grown to an OD_600_ of ∼1.8 in broth. For induction of the inducible pSIP411-OVA_f_ plasmid, 20 ng/ml SppIP (Molecular Biology Unit, University of Newcastle, UK) was added to bacterial cultures at OD_600_ ∼0.3. Bacteria were harvested by centrifugation, washed twice in endotoxin-free Dulbecco’s PBS (PAA Laboratories, Linz, Austria), adjusted to 2×10^9^ bacteria/ml by counting under the microscope. To prevent bacterial replication inside APCs, the bacteria were inactivated by UV-irradiation for 18 min. The effectiveness of this treatment in killing the bacteria was confirmed by lack of bacterial growth after overnight incubation on blood agar.

Soluble OVA (Sigma) was purified of contaminating LPS using Detoxi-gel (Pierce, Rockford, IL). After this treatment, <5 EU of LPS was present in 100 µg OVA (Chales River Endosafe test, Coatech, Kungsbacka, Sweden).

### Preparation and Analysis of Antigen-presenting Cells

BALB/c mice (B&K, Sollentuna, Sweden) were sacrificed at 6–12 weeks of age. Single cell spleen suspensions were prepared and erythrocytes were lysed (0.15 M NH_4_Cl, pH 7.3, for 5 min at 37°C). CD11c^+^ cells (DCs) were enriched using α-CD11c-coated magnetic MACS beads (Miltenyi Biotec, Bergisch Gladbach, Germany) and LS-columns (Miltenyi Biotec) according to the manufacturer’s instructions. In some cases, CD11c^+^ DCs were further purified by FACS-sorting (FACSAria, BD, San José, CA) using a 100 µm nozzle.

APCs were analyzed by flow cytometry (FACSCanto, BD Biosciences, Treestar, Ashland, OR) after pre-incubation with an FcγR-blocking mAb and staining with antibodies against CD11c, B220, CD11b, Ly6C, MHCII, CD8α or appropriate isotype controls (BD Pharmingen, San Diego, CA).

### Co-culture of Spleen APCs and OVA-specific T cells

OVA-specific T cells were purified from OVA-TCR-transgenic DO11.10 mice, whose CD4^+^ T cell receptor recognizes the immunodominant epitope OVA_323–339_ [21]. Mice were sacrificed at 6–22 weeks of age, and the lymph nodes were excised. T cells carrying the transgenic T cell receptor were isolated using FITC-conjugated anti-DO11.10 TCR antibody (KJ1-26) [22], followed by α-FITC-conjugated MACS beads (Miltenyi Biotec). The isolation routinely resulted in >85% CD4^+^ KJ1-26^+^ cells.

Unfractionated or CD11c-purified BALB/c splenocytes were used as APCs. APCs were suspended in Iscove’s medium supplemented with 10% FCS, 1% L-glutamine, 1% mercaptoethanol, and 0.01% gentamycin (all from Sigma), and pulsed with UV-inactivated OVA-producing bacteria or control bacteria (5×10^6^/ml or 5×10^7^/ml) or with soluble endotoxin-free OVA (10 µg/ml or 100 µg/ml). For use of unfractionated spleen cells as APCs, BALB/c spleen single cell suspensions (2.5×10^6^/ml) were pulsed with bacteria or OVA for 18 h, irradiated (2,500 Rad), washed, and aliquoted at 2.5×10^6^ cells/ml in 96-well round-bottom plates (Nunc). CD11c^+^ cells were used at 1×10^5^/ml (MACS-enriched CD11c^+^ cells) or 2.5×10^4^/ml (FACS-purified CD11c^+^ cells) and were pulsed with bacteria or OVA for 2 h in 96-well round-bottom plates, centrifuged in the plates, and washed ×3 with medium.

OVA-specific T cells were added to the antigen-pulsed APCs at 2.5×10^5^ cells/ml. After 4 d, supernatants were collected and proliferation was measured by [^3^H]-thymidine incorporation (Amersham, Arlington Heights, IL). Supernatant IFN-γ, IL-17, IL-10, and IL-13 concentrations were determined using DuoSet kits (R&D Systems, Minneapolis, MN) according to the manufacturer’s instructions.

### Assessment of Phagocytosis

Phagocytosis of bacteria was assessed using GFP-transformed bacteria or pHrodo™ succinimidyl ester (Invitrogen) stained bacteria (0.5 mM pHrodo was used and staining was performed according to the manufacturer’s instructions). The pHrodo dye was used to assess presence of bacteria in phagolysosomes, since the pH-sensitive dye emits fluorescence only in acidic environments. The APCs were incubated with fluorescent bacteria (5×10^7^/ml) for 50 min at 37°C in 24-well plates (Nunc, Roskilde, Denmark). The plates were placed on ice, whereafter cells were detached using a rubber policeman, fixed in 1% paraformaldehyde (Sigma), stained with APC-conjugated α-CD11c and PerCp-conjugated α-CD11b (BD Pharmingen) and analysed by flow cytometry. Gated CD11c^+^SSC^low^ cells that showed higher fluorescence intensities in the FL1 channel than the empty FL2 channel were regarded to be associated with GFP-producing bacteria, while cells with higher fluorescence intensities in the FL2 channel than in the FL1 (empty) channel were regarded to have internalised the pHrodo-stained bacteria.

### APC Activation Marker Expression and Cytokine Production after Interaction with Bacteria

MACS-enriched CD11c^+^ cells (1×10^5^/ml), or unfractionated cells (2–2.5×10^6^/ml), were stimulated with *E. coli* or *Lactobacillus sakei* overnight. The levels of IL-12, TNF, IL-10, and IL-6 in the culture supernatants were quantified using the Cytometric Bead Array (BD Pharmingen), PGE_2_ was measured using an EIA monoclonal kit (Cayman Chemical Company, Ann Arbor, MI), and IL-1β was measured using a DuoSet kit (R&D Systems, Minneapolis, MN). CD11c^+^ cells were analysed by flow cytometry for the expression of CD86 and CD40.

### Statistical Analysis

Student’s *t*-test was employed for comparisons of T cell proliferation and cytokine production in response to different stimuli and in response to splenic *vs* peritonal APCs as well as to compare mediator production by stimulated splenic and peritoneal cells. Student’s paired *t*-test was employed to compare proliferation and cytokine production in response to OVA-expressing and non-OVA-expressing control bacteria. All reported p-values are two-sided.

## Results and Discussion

### OVA-expressing *E. coli* Activate OVA-specific T cells more Efficiently than does Soluble OVA

**Figure 1:**
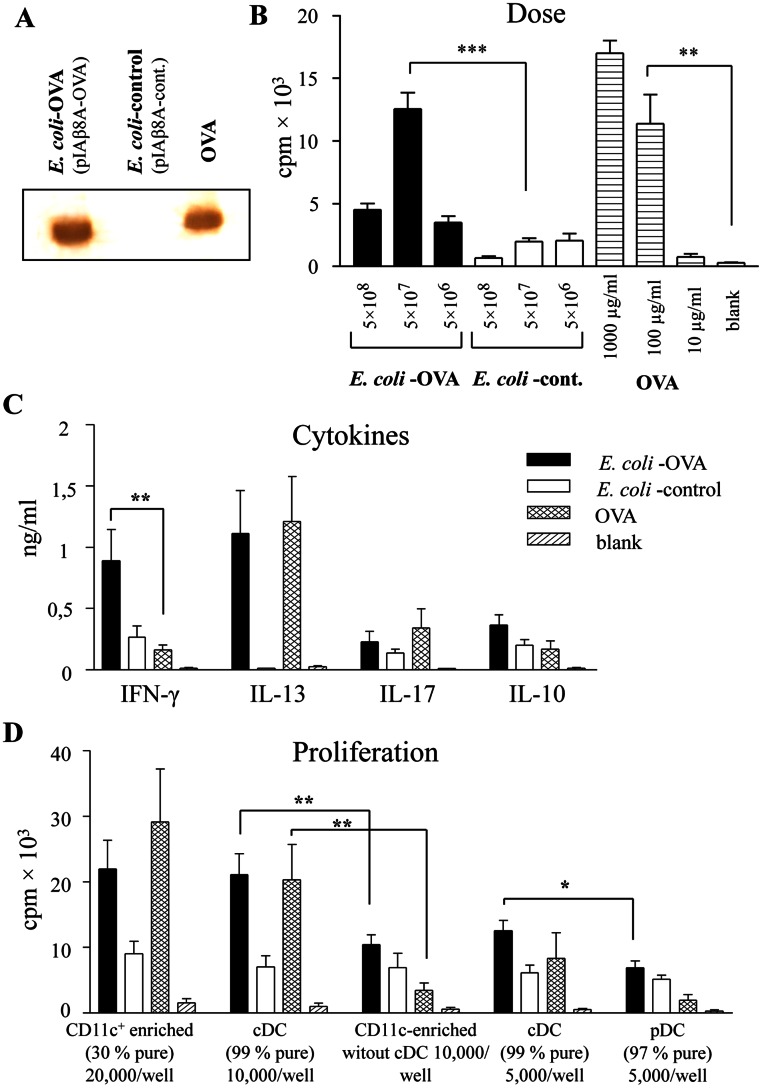
OVA-specific T-cell responses to OVA produced intracellularly in *E. coli*. *E. coli* HB101 was transformed with the pIAβ8A-OVA plasmid encoding full-length ovalbumin (OVA), or an empty pIAβ8A-control plasmid. A, Western blot showing presence of OVA in sonicates of the transformed bacteria; OVA = soluble OVA standard. B, Proliferative responses of OVA-specific transgenic DO11.10 T cells co-cultured for 4 d with irradiated splenocytes pulsed with *E. coli*-OVA, *E. coli*-control or soluble OVA (n = 6 mice for 5×10^7^/ml bacteria and 100 µg/ml OVA; n = 2 for the other concentrations). C, Production of cytokines in 4 d co-cultures of OVA-specific T cells and CD11c^+^-enriched splenocytes pulsed with bacterial or soluble OVA (n = 6). D, Proliferative responses by 4 d co-cultures of OVA-specific T cells and APCs pulsed with bacterial or soluble OVA. The APCs used were CD11c^+^ MACS-bead enriched cells (CD11c^+^ enriched), or cells further purified by FACS-sorting into CD11c^+^B220^−^ cells (cDCs) CD11c^low^B220^+^Ly6C^+^CD19^−^ cells (pDC) or cells lacking CD11c^+^B220^−^ (CD11c^+^ enriched cells without cDCs) (n = 6 for CD11c^+^-enriched cells and cDCs; n = 4 for other APCs). * *P*<0.05; ** *P*<0.01; *** *P*<0.001.

A plasmid encoding full-length OVA protein (pIAβ8A-OVA) was introduced into *E. coli* HB101 (see *Materials and Methods*). Production of OVA was confirmed by Western blotting of the sonicated bacteria (Fig. 1A). Quantification by ELISA revealed that a sonicate of 5×10^7^ OVA-transformed bacteria contained 0.19 µg of OVA. The culture supernatant contained 100 times less OVA than the culture sonicate, demonstrating that *E. coli*-produced OVA was almost exclusively intracellular.

Unfractionated splenic APCs were pulsed for 18 h with various doses of washed and UV-killed bacteria containing OVA (*E. coli*-OVA), corresponding *E. coli* control bacteria, or soluble OVA (purified and LPS-free), after which the cells were irradiated, washed and co-cultured with OVA-specific DO11.10 CD4^+^ T cells. OVA-specific proliferation was calculated as the proliferative responses to the OVA-containing *E. coli* minus that induced by control *E. coli* (Δprol). Maximal proliferation was observed after 4–5 days of co-culture in response to both “bacterial” and soluble OVA (data not shown). Regarding antigen concentration, OVA-expressing *E. coli* induced maximal T-cell proliferation at 5×10^7^/ml (Δprol = 12,000 cpm), while the response to the same concentration of *E. coli-*control was negligible (Fig. 1B). Soluble OVA at a concentration of 100 µg/ml induced a comparable proliferative response as 5×10^7^/ml OVA-producing *E. coli* (Fig. 1B). Since 5×10^7^ bacteria contained only 0.19 µg OVA, 500-fold less *E. coli*-produced OVA than soluble OVA was needed to obtain the same OVA-specific T cell proliferative response.

Bacteria might be strong immunogens both because they are particles and because they express danger signals. To investigate if OVA needed to be contained within intact *E. coli* to be strongly immunogenic, OVA-producing *E. coli* were sonicated before being used in APC-T cell co-cultures. Sonication of *E. coli*-OVA reduced their capacity to trigger OVA-specific T cell proliferation, compared with intact *E. coli*-OVA (Fig. S2). However, sonicated *E. coli*-OVA were still more immunogenic than the same dose of soluble OVA. We cannot exclude that the sonicate still contained quite large bacterial fragments with associated or trapped OVA. LPS and other cell wall components may also induce maturation of DCs, enabling them to more efficiently present soluble OVA. However, this might be less likely, considering the minute amounts of OVA present in the bacteria.

The spleen contains several potential APC populations including conventional and plasmacytoid DCs, monocytes, macrophages and B cells. Conventional CD11c^+^ DC are regarded as the most efficient APC [23]. We enriched CD11c^+^ cells to ∼30% purity using α-CD11c-coated magnetic beads, or to >99% by flow cytometry sorting, pulsed them for 2 h with *E. coli-*OVA, *E. coli-*control, or soluble OVA and assessed their ability to stimulate OVA-specific T cells. The CD11c^+^ cells supported strong T-cell proliferation in response to both *E. coli*-OVA and soluble OVA, suggesting that these cells were responsible for the major APC function of spleen cells (Fig. 1D). In contrast, FACS-sorted CD11c^int^B220^+^Ly6c^+^ plasmacytoid DCs loaded with soluble or bacterial OVA were inefficient at activating OVA-specific T cell proliferation (Fig. 1D).

The bacterial carrier of an antigen may, in addition to boosting proliferation, also affect whether the naïve T cells will differentiate into Th1, Th2, Th0 (mixed Th1 and Th2), or Th17 cells. We investigated signature cytokines for these T cell subsets in T cell-APC co-cultures. T cells activated by APCs pulsed with soluble OVA produced foremost the Th2 cytokine IL-13 (Fig. 1C). In contrast, T cells responding to *E. coli*-OVA produced high levels of both IFN- γ and IL-13, suggesting a balanced Th1/Th2 response, sometimes referred to as Th0 [24] (Fig. 1C). The T cell cytokine pattern was very similar regardless of whether unfractionated spleen cells or CD11c^+^-enriched splenocytes were used as APCs (data not shown).

### OVA Expressed within *E. coli* Induces Stronger OVA-specific CD4^+^ T-cell Proliferation than OVA Expressed within Lactobacilli or Lactococci

Next, we asked whether T cell activation and polarisation would differ if OVA would be expressed in G+ bacteria, rather than the G− *E. coli*. The pIAβ8A-OVA vector used to transform *E. coli* did, however, not support OVA expression in the G+ bacteria tested (data not shown). Not only do G+ and G− bacteria require different expression plasmids, but they also use different codons for the same amino acid. To allow for comparison of T cell responses to G+ and G− bacteria, we synthesised a gene, *OVA_f_*, encoding the amino acids 319–386 of the OVA protein but with codons optimised for expression in lactobacilli (Fig. S1). The *OVA_f_* gene, which contains the epitope recognised by DO11.10 T cells (a.a. 323–339), was inserted into pSIP401-based inducible or constitutive expression plasmids suitable for G+ bacteria. These constructs were transformed into the G+ lactobacilli *L. sakei* Lb790 and *L. plantarum* NC8, the lactococcus strain *L. lactis* MG1363, as well as the G− *E. coli* XL-10. A synthetic *GFP* gene optimised for lactobacilli with regard to codon usage (Axelsson, L., unpublished) was inserted into the same plasmids, and bacteria transformed with these plasmids were utilised as OVA-negative control bacteria and for analysis of bacterial uptake by phagocytosis.

**Figure 2:**
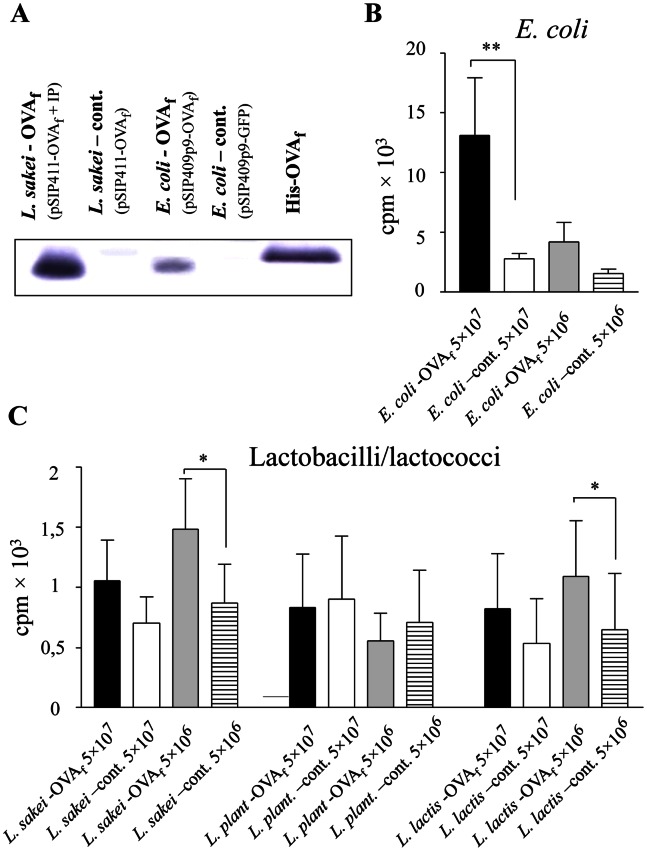
OVA-specific T-cell responses to OVA fragment 319–386 produced intracellularly in *E. coli,* lactobacilli and lactococci. *Lactobacillus sakei*, *Lactobacillus plantarum*, *Lactococcus lactis*, and *E. coli* were transformed with vectors encoding an immunodominant OVA fragment (OVA_f_). A, Western blot showing production of OVA_f_ by *L. sakei* and *E. coli*; expression is lower in *E. coli* due to use of a vector optimal for G+ bacteria. His-tagged OVA_f_ was used as a molecular mass marker (one representative experiment of three). Proliferative responses of DO11.10 T cells stimulated with irradiated splenocytes pulsed with OVA_f_-transformed *E. coli (B)*, or lactobacilli/lactococci (C). Despite lower expression of OVA_f_ in *E. coli*, OVA-specific proliferation is much more pronounced in cultures with transformed *E. coli* than in cultures with transformed lactobacilli/lactococci; note the different scales in the respective graphs (n = 5 for *E. coli* and *L. sakei*; n = 3 for *L. plantarum* and *L. lactis*). * *P*<0.05; ** *P*<0.01.

Semiquantitative analysis of *OVA_f_* production by Western blotting and quantification of GFP expression by fluorescence scanning of bacterial sonicates showed that both the inducible pSIP411-OVA_f_ vector and the constitutive pSIP411p9-OVA_f_ or -GFP vectors supported high-level expression of *OVA_f_*_,_ as well as of GFP, in *L. sakei* and *L. plantarum*, but lower protein expression in *L. lactis* and *E. coli* (Fig. 2A, Table 2). This was expected, as both the vectors and the gene codon usage were optimized for expression in lactobacilli.

**Table 2:** Relative expression levels of OVA fragment and GFP by transgenic Gram-negative and Gram-positive bacteria.

Strains	Plasmid	Relative OVA-expression	GFP-expression
*Escherichia coli* XL10 Gold	pSIP409p9-OVA_f_	+	0
	pSIP409p9-GFP	−	0.11
*Lactobacillus sakei* Lb790	pSIP411-OVA_f_+IP	+++	0
	pSIP411-OVA_f_	+/−	0
	pSIP411p9-OVA_f_	++	0
	pSIP411p9-GFP	−	1.0
*Lactobacillus plantarum* NC8	pSIP411-OVA_f_+IP	+++	0
	pSIP411-OVA_f_	+/−	0
	pSIP411p9-OVA_f_	++	0
	pSIP411p9-GFP	−	1.0
*Lactococcus lactis* MG1363	pSIP411p9-OVA_f_	+	0
	pSIP411p9-GFP	−	0.08

*E. coli* and three G+ bacterial strains were transformed with plasmids carrying a synthetic gene that encodes amino acids 319–386 of chicken ovalbumin (termed OVA fragment; OVA_f_), or green fluorescent protein (GFP). After sonication of the bacteria, the amount of OVA_f_ was determined in a semiquantitative manner by examination of Western blots by the naked eye: +/− very low,+low,++intermediate,+++strong expression. The amount of GFP was determined by scanning two-fold serial dilutions of bacterial sonicates in a Typhoon 8600 Imager. The GFP-expressing bacteria giving the highest fluorescence emisson was set to 1.0.

G+ and G− bacteria carrying an expression plasmid for the immunodominant OVA fragment *OVA_f_* were UV-inactivated, washed and fed to unfractionated spleen APCs. After 18 h, APCs were washed, irradiated and incubated with OVA-specific DO11.10 T cells (Fig. 2B–C). Despite the suboptimal construction of the plasmids for use in G− bacteria, *E. coli* XL-10 transformed with the *OVA_f_* -plasmid induced strong OVA-specific T-cell proliferation (Fig. 2B). In fact, the response was of similar magnitude as that induced by *E. coli* HB101 expressing the full-length *OVA* gene (Δprol = 12,000 cpm for 5×10^7^ bacteria/ml) (Fig. 2B, compare with Fig. 1C). To the contrary, both lactobacilli and lactococci transformed with *OVA_f_*, were strikingly less efficient stimulators of OVA-specific T-cell proliferation than *OVA_f_* transformed *E. coli* (note the different scales in the graphs in Fig. 2B and 2C). Thus, *L. sakei* transformed with the inducible OVA-vector induced a significant, but weak, OVA-specific T-cell proliferation (Δprol = 400 cpm for 5×10^7^ bacteria/ml, and Δprol = 600 cpm for 5×10^6^ bacteria/ml). An OVA-specific response of similar magnitude was induced by *OVA_f_* presented in *Lactococcus lactis*, while no OVA-specific T cell proliferation was seen when APCs were pulsed with *OVA_f_*- transformed *Lactobacillus plantarum* (Fig. 2C). This was not due to different kinetics of the response to G+ and G− bacteria, as T cell proliferation in response to *L. sakei*-OVA and *L. lactis*-OVA was maximal day 4–5, as was the response to soluble and *E. coli*-expressed OVA. Furthermore, no response to *L. plantarum*-OVA was seen at any of the time-points tested (Days 1–7).

**Figure 3:**
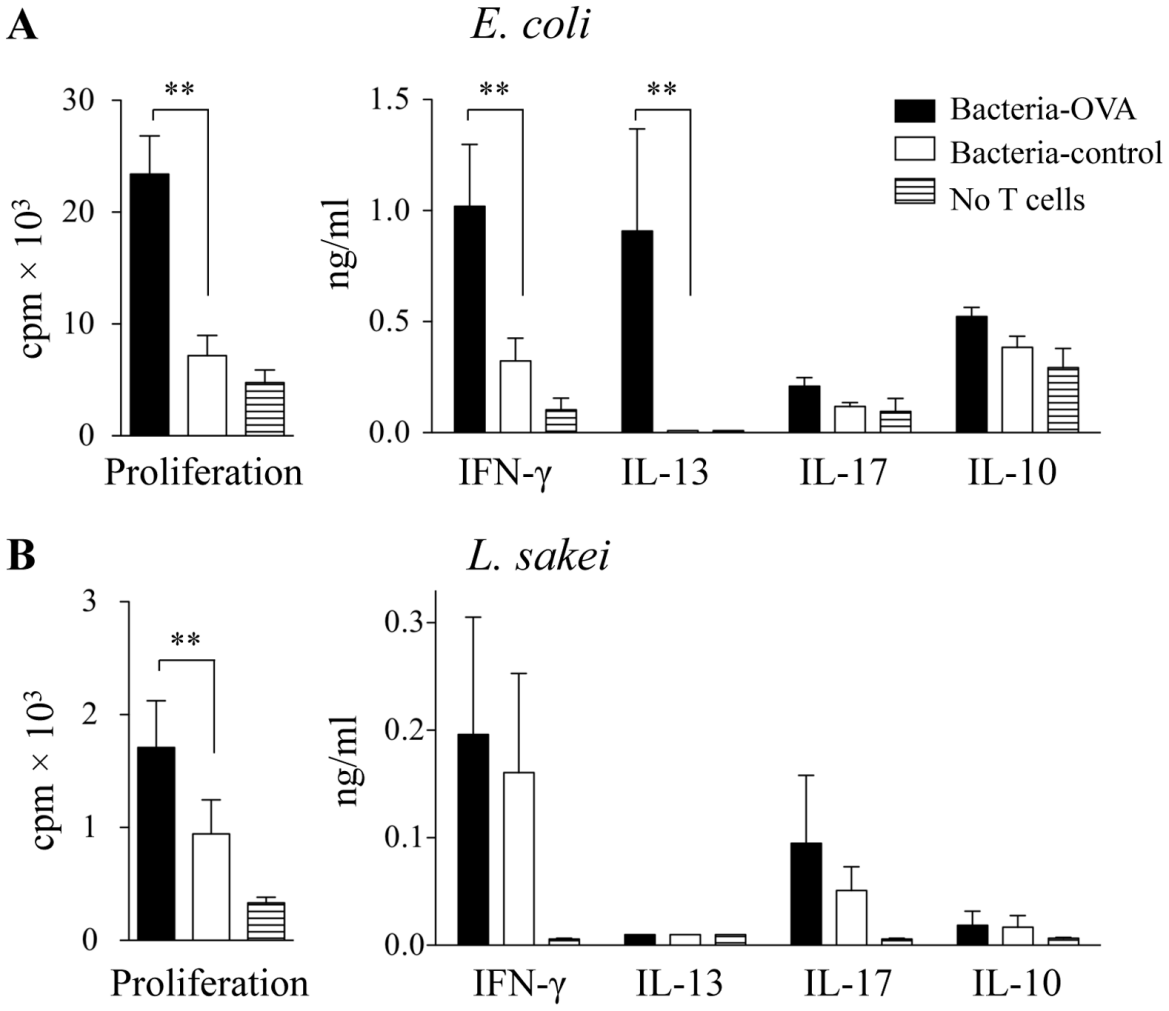
T cell proliferation and cytokine production in response to *E. coli* and *L. sakei* expressing OVA_f_. Proliferative response and cytokine production by 4 d co-cultures of OVA-specific DO11.10 T cells and CD11c^+^-enriched splenocytes pulsed with OVA_f_-expressing *E. coli* (A) or *L. sakei* (B) or the respective control bacteria transformed by an empty plasmid (n = 5). ** *P*<0.01.

The ability of transformed G+ bacteria to induce T cell proliferation did not relate to the amount of OVA produced by the strain in question. Hence, both lactobacillus strains (*L. sakei* and *L. plantarum*) produced high levels of intracellular *OVA_f_*, but *L. sakei* induced a measurable OVA-specific response, while *L. plantarum* did not. Furthermore the lactococcus strain (*L. lactis*) produced less OVA than did the other G+ strains (Table 2), but induced a similar OVA-specific response as did *L. sakei*. Feeding more G+ bacteria to the APCs did not compensate for the poor immunogenicity, as two of the G+ strains were even less stimulatory at 5×10^7^ bacteria/ml than at 5×10^6^ bacteria/ml (Fig. 2C). At 5×10^5^ bacteria/ml no OVA-specific T cell proliferation was recorded (data not shown). Similar results were obtained when using CD11c-enriched splenocytes as APCs (Fig. 3 and data not shown).

We also assessed cytokine responses of DO11.10 cells exposed to APCs pulsed with OVA-producing G+ bacteria. *L. sakei* and *L. lactis* transformed with *OVA_f_* encoding plasmid induced some IFN-g and IL-17, but no detectable IL-13 or IL-10 production (Fig. 3B and data not shown). *OVA_f_* expressing *E. coli* XL10 induced a mixed Th1/Th2 response dominated by IFN-g and IL-13, similar to the response seen to full-length OVA expressed by HB101 (Fig. 3A).

### Role of Phagocytosis and APC Maturation

We investigated whether poor phagocytosis was the cause of the inefficient presentation of OVA expressed intracellularly in G+ bacteria. UV-inactivated *E. coli* and *L. sakei* were stained with pHrodo, which emits fluorescence in acidic environments, such as that within the phagolysosome. Splenic DCs were allowed to interact with the labelled bacteria for 50 min, whereafter fluorescence was measured. Fluorescent *E. coli* were detected in 0.98±0.08% of the CD11c^+^SSC^low^ DCs, while *L. sakei* was present in 3.1±0.6% of the DCs. Similar results were obtained using flow cytometric analysis of GFP-expressing bacteria (not shown). As *L. sakei* was readily phagocytosed, inefficient uptake could not explain the low immunogenicity of the G+ bacteria.

Expression of co-stimulatory molecules on the APC surface and APC production of T cell stimulating cytokines (including IL-12) are central in stimulating T cell proliferation and maturation. The capacity of G− (*E. coli*) and G+ (*L. sakei*) bacteria to induce APC cytokines and co-stimulatory molecules was compared.

**Figure 4:**
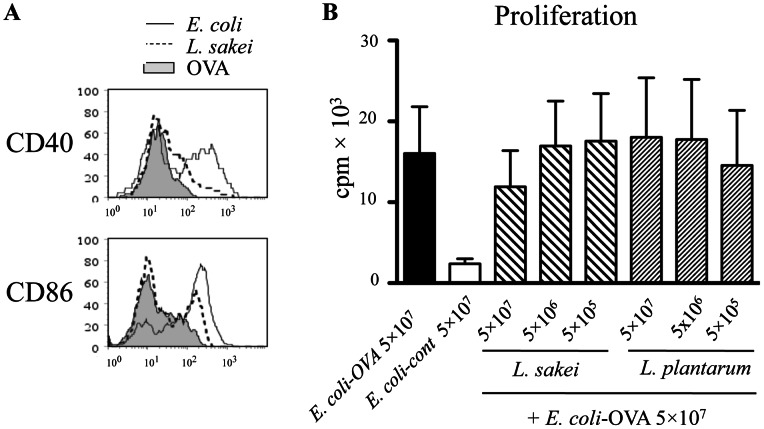
Bacteria trigger expression of activation markers on APCs, and the lactobacilli do not reduce the T cell stimulatory capacity of DCs. A, Splenocytes were stimulated overnight with 5×10^7^/ml of UV-inactivated *L. sakei* or *E. coli* and the expression of CD40 and CD86 on CD11c^+^ DCs was determined by flow cytometry. The results of one representative experiment of three performed are shown. B, CD11c^+^-enriched splenocytes were pulsed with 5×10^7^/ml UV-inactivated *E. coli*-OVA (or *E. coli*-control) in the absence or presence of graded doses of UV-inactivated *L. sakei* or *L. plantarum.* The antigen-presenting cells were thereafter co-cultured with OVA-specific DO11.10 T cells and proliferation was measured day 5 (n = 5).

*E. coli* and *L. sakei* both triggered upregulation of CD86 and CD40 on DCs, but considerably higher levels were induced by *E. coli*; this was particularly evident for CD40 (Fig. 4A). This is in accordance with previous studies where G− bacteria have been shown to induce DC maturation more efficiently than G+ bacteria [12], [13]. This has been linked to the ability of LPS to simultaneously activate MyD88- and TRIF-dependent pathways within DCs [25]. Exposure to LPS-free OVA did not induce any expression of co-stimulatory molecules (CD86, CD40) on DCs.

We investigated whether exposure to LPS could enhance the T cell stimulating capacity of APCs that had phagocytosed OVA-expressing G+ bacteria. Addition of LPS to the co-cultures resulted in an increased non-OVA-specific proliferation, but did not significantly enhance the OVA-specific T cell proliferation to *L. sakei-*OVA (data not shown). Hence, lack of DC activation could not be the only explanation to the poor immunogenicity of *L. sakei*, *L. lactis* and *L. plantarum*, compared to *E. coli*. We also addressed the possibility that G+ bacteria might actively counteract the ability of APCs to present antigens to T cells. Thus, splenic DCs were pulsed with 5×10^7^ *E. coli-*OVA/ml in the presence or absence of graded doses of *L. sakei*, or *L. plantarum*. The G+ bacteria did not significantly alter the OVA-specific T cell activation in response to *E. coli-*OVA (Fig. 4B).

Next, splenic APCs were tested for production of cytokines in response to overnight incubation with *E. coli* and *L. sakei*. As seen in Table 3, *L. sakei* induced at least as much IL-12, TNF and IL-6 as did *E. coli*. Stimulation with *E. coli* induced a higher production of IL-10 than *L. sakei* (Table 2), but since IL-10 would rather counteract T cell activation [26], the presence of IL-10 cannot explain the higher T cell stimulatory capacity of APCs pulsed with G− than G+ bacteria. Exposure of splenocytes to soluble OVA for 16 h did not result in the production of any of the investigated cytokines (IL-12, TNF, IL-6). Since soluble OVA, that did not activate APCs, induced IL-13 producing T cells, one may speculate that Th2 polarization is the default T cell maturation pathway induced by non-activated splenic APC. In contrast, when cytokines such as IL-12 and TNF and co-stimulatory molecules are induced by bacterial stimulation, this favours Th1 maturation and production of IFN-. In accordance, bacteria induced maturation of monocyte derived DC has been shown to favour Th1 polarisation to presented unrelated antigens [13], [14], [15].

**Table 3:** Cytokine production by bacteria-stimulated splenocytes (n = 5, mean ± SEM).

	IL-12 (pg/ml)	TNF (pg/ml)	IL-10 (pg/ml)	IL-6 (ng/ml)
*E. coli*	14±4	580±80	670±160*	0.47±0.06
*L. sakei*	64±40	830±180	34±20	0.40±0.09

* *P*<0.05; significant difference in mediator production by splenic cells stimulated with either *E. coli* (5×10^7^) or *L. sakei* (5×10^6^).

From our results it seems that poor uptake could not explain the inefficient presentation of an intracellular protein in G+ bacteria and that lack of LPS might contribute, but not fully explain this defect. An alternative explanation may be inefficient loading of peptides on MHC II molecules. The G+ cell wall is much thicker (>50 layers of peptidoglycan), more sturdy and tighter meshed than the cell wall of G− bacteria, which often have a single sparsely cross-linked peptidoglycan layer. Break-down of G+ bacteria in the phagolysozome is therefore a more demanding task than lysis of G− bacteria. Thus, the rigid cell wall of G+ bacteria may delay the release of OVA in the APC after internalization. One may also speculate that, compared to easily digestible G− bacteria, the presence of poorly digestible G+ bacteria within the phagosome triggers a stronger lysosomal maturation, the process whereby lysozyme (which degrades cell wall peptidoglycan), proton pumps and bacteria-degrading enzymes are recruited into the phagosome. As it takes longer time to digest a G+ than a G− bacterium, higher concentrations of proteolytic enzymes may accumulate, resulting in a more extensive degradation of bacterial proteins, including OVA. Thus, in the highly proteolytic environment, the peptides from Gram-positive bacteria may become too degraded for loading onto MHC II. In support of the theory of inefficient loading of proteins present within bacteria with a rigid cell wall, the cell wall of *L. plantarum* is more resistant to digestion with lysozyme, compared to those of *L. sakei* and *L. lactis* (Axelsson, L., unpublished observation). Despite being produced in high levels, OVA expressed within *L. plantarum* was presented least efficiently by APCs to T cells. Notably, pulsing APCs with sonicates of G+ bacteria did not induce any OVA-specific T cell activation (unpublished observations), suggestedly because soluble bacterial constituents are taken up less efficiently than intact bacteria. In addition, sonicated G+ bacteria have been shown to trigger less APC activation, compared to intact G+ bacteria [27].

In summary, we demonstrate a striking difference in efficiency of antigen presentation to CD4^+^ T cells when an antigen (OVA) was present in its soluble form or expressed in G− or G+ bacteria. The expression of OVA in *E. coli* led to efficient immunogenic presentation to OVA-specific T cells, hence 500-fold less “bacterial OVA” than soluble OVA was needed to induce the same magnitude of T cell proliferation. We could also demonstrate a shift in T cell maturation pathways, from a Th2 to a mixed Th1/Th2 phenotype when DCs had been incubated with OVA expressed within *E. coli*, compared to soluble OVA. Despite producing large amounts of OVA and despite being efficiently phagocytosed, G+ bacteria were, compared with G− bacteria, inefficient vehicles for the delivery of immunogenic protein to antigen-presenting cells. The results suggest that intracellular expression of proteins within G+ bacteria conveys poor immunogenicity, which may be taken into account when considering G+ bacteria as vehicles for vaccine delivery.

## Supporting Information

Figure S1 A, The amino acid (a.a.) sequence of the C-terminal peptide (a.a. 319–386) of chicken ovalbumin. B, Sequence of the gene for chicken ovalbumin (a.a. 319–386). C, Sequence of the gene for the synthetic ovalbumin (a.a. 319–386) with codons optimised for lactobacilli codon usage (CAI = 0.846). The gene also contains the methionine start codon (atg) and restriction sites for *Nco*I, *Xba*I, and *Xho*I.
(DOCX)

Figure S2 Sonicated *E. coli*-OVA are less immunogenic than intact bacteria. Proliferative response after 5 d co-culture of OVA-specific DO11.10 T cells and CD11c^+^-enriched splenocytes pulsed with intact or sonicated *E. coli*-OVA. Bars show mean+SEM proliferation induced by APCs from 2 mice.
(TIF)

Methods S1 Construction of OVA-expression plasmids.
(DOCX)
