# Supplementary material for: Stronger T Cell Immunogenicity of Ovalbumin Expressed Intracellularly in Gram-Negative than in Gram-Positive Bacteria
Source: PLoS One. 2013 May 31;8(5):e65124. doi: 10.1371/journal.pone.0065124 (PMC3669294; doi:10.1371/journal.pone.0065124)
Supplement: Figure S1 — A, The amino acid (a.a.) sequence of the C-terminal peptide (a.a. 319–386) of chicken ovalbumin. B, Sequence of the gene for chicken ovalbumin (a.a. 319–386). C, Sequence of the gene for the synthetic ovalbumin (a.a. 319–386) with codons optimised for lactobacilli codon usage (CAI = 0.846). The gene also contains the methionine start codon (atg) and restriction sites for NcoI, XbaI, and XhoI. (DOCX) [file pone.0065124.s001.docx]

**A**, C-terminal peptide (amino acids 319-386) of chicken ovalbumin:

AESLKISQAVHAAHAEINEAGREVVGSAEAGVDAASVSEEFRADHPFLFCIKHIATNAVLFFGRCVSP

**B**, Original gene sequence encoding amino acids 319-386 of ovalbumin

5′-GCAGAGAGCCTGAAGATATCTCAAGCTGTCCATGCAGCACATGCAGAAATCA

ATGAAGCAGGCAGAGAGGTGGTAGGGTCAGCAGAGGCTGGAGTGGATGCTGCAAGCGTCTCTGAAGAATTTAGGGCTGACCATCCATTCCTCTTCTGTATCAAGCACATCGCAACCAACGCCGTTCTCTTCTTTGGCAGATGTGTTTCCCCTTAA-3′

**C**, Sequence of the synthetic gene that encodes amino acids 319-386 of ovalbumin.

5′-tag**ccatgG**CTGAATCATTGAAAATCTCACAAGCAGTTCACGCTGCTCACGCTGAA

ATCAACGAAGCAGGTCGTGAAGTAGTTGGATCTGCTGAAGCTGGTGTTGACGCTGCTTCAGTTTCAGAAGAATTTCGTGCAGATCATCCATTCTTGTTCTGTATTAAACACATCGCTACTAACGCAGTTCTTTTCTTCGGTCGTTGTGTATCTCCATAAtaatat**tctagactcgag**tat-3′
