# Supplementary figures and images for: Stronger T Cell Immunogenicity of Ovalbumin Expressed Intracellularly in Gram-Negative than in Gram-Positive Bacteria
Source: PLoS One. 2013 May 31;8(5):e65124. doi: 10.1371/journal.pone.0065124 (PMC3669294; doi:10.1371/journal.pone.0065124)

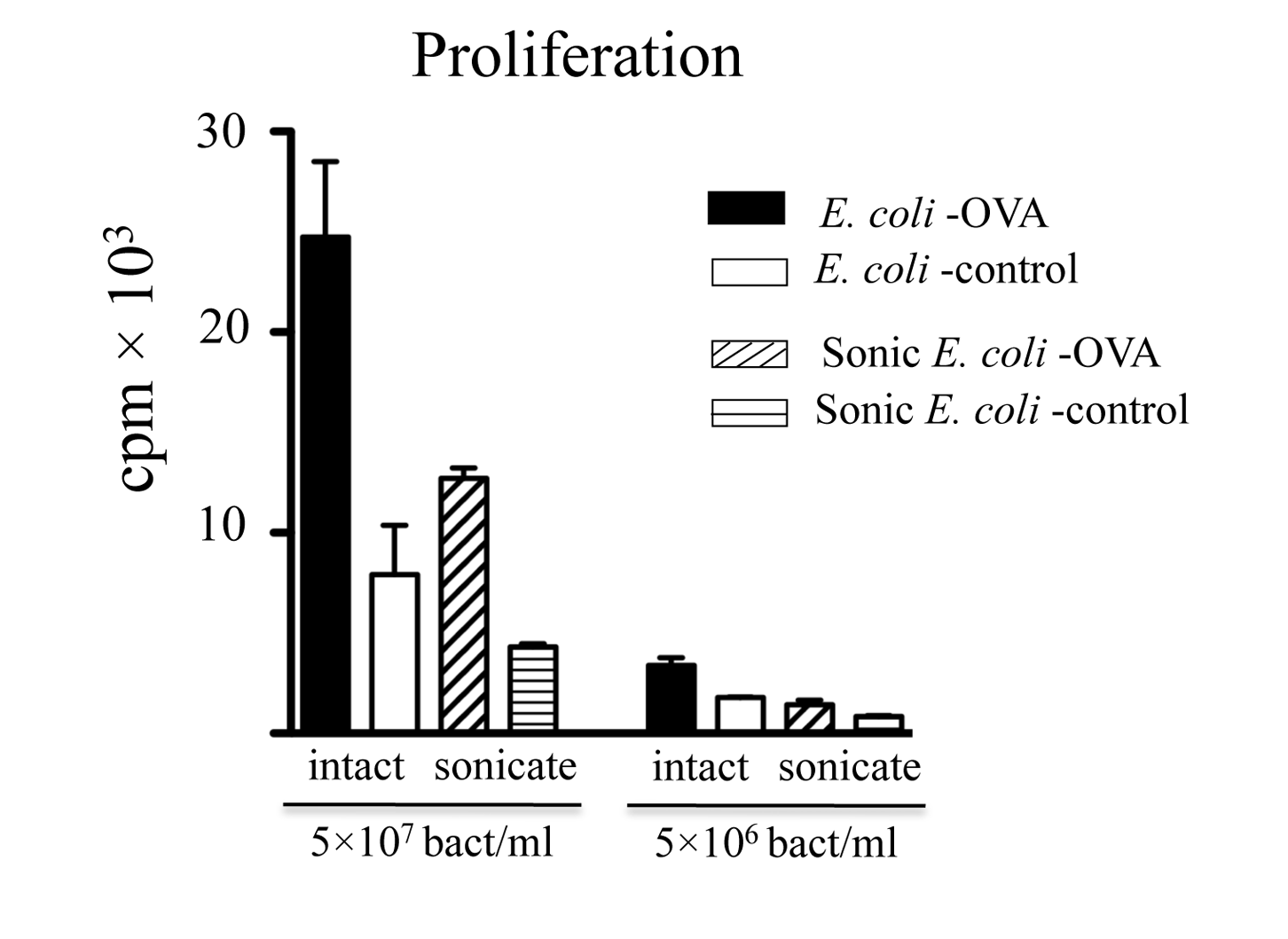

Supplement: Figure S2 — Sonicated E. coli-OVA are less immunogenic than intact bacteria. Proliferative response after 5 d co-culture of OVA-specific DO11.10 T cells and CD11c+-enriched splenocytes pulsed with intact or sonicated E. coli-OVA. Bars show mean+SEM proliferation induced by APCs from 2 mice. (TIF) [file pone.0065124.s002.tif]
