# Supplementary material for: Stronger T Cell Immunogenicity of Ovalbumin Expressed Intracellularly in Gram-Negative than in Gram-Positive Bacteria
Source: PLoS One. 2013 May 31;8(5):e65124. doi: 10.1371/journal.pone.0065124 (PMC3669294; doi:10.1371/journal.pone.0065124)
Supplement: Methods S1 — Construction of OVA-expression plasmids. (DOCX) [file pone.0065124.s003.docx]

**METHODS S1**

**Construction of *E. coli* that produces the full-length OVA protein**

An OVA-expressing plasmid was constructed by combining elements of the plasmids pOMP21 [[1](#_ENREF_1),[2](#_ENREF_2)] and pIAβ8 [kindly supplied by Professor Perez-Martinez [[3](#_ENREF_3)]]. pOMP21 is a constitutive *lac* gene-based *E. coli* expression vector that contains the cDNA for ovalbumin (amino acids 5-386) fused in phase with the seventh amino acid of β-galactosidase [[1](#_ENREF_1),[2](#_ENREF_2)]. The shuttle plasmid pIAβ8 replicates in both Gram-negative and Gram-positive bacteria [[3](#_ENREF_3)]. Both pIAβ8 and pOMP21 contain the *lac* operon, which was eliminated from pIAβ8 by *Sca*I digestion, resulting in pIAβ8A. The OVA-encoding fragment and the lac operon were removed from pOMP21 by digestion with *Hha*I, gel purified (Geneclean II kit; Q-Biogene, Irvine, CA), blunted, ligated into the *Sma*I site of the pIAβ8A plasmid, and transformed into *E. coli* HB101. HB101 bacteria that contained the pIAβ8A plasmid without the *OVA* gene were used as “empty” control bacteria.

**Construction of recombinant lactobacilli, lactococci, and *E. coli* that produce a synthetic OVA fragment or GFP**

A synthetic gene that encodes amino acids 319-386 of chicken ovalbumin (i.e., the *OVA* fragment; OVA_f_), and which was adapted with respect to the codon usage of lactobacilli (Suppl. Fig. 1), was generated. The gene was flanked by the restriction sites *Nco*I (start codon) and *Xho*I, and was inserted into the multiple cloning site of the pUC57 plasmid (GeneScripts Corp., Piscataway, NJ).

The 224-bp OVA_f_ was digested with *Nco*I and *Xho*I, gel purified (Qiagen), ligated into the *Nco*I and *Xho*I sites of the pSIP411 vector, and electrotransformed into *L. plantarum* NC8 and *L. sakei* Lb790 as described [[4](#_ENREF_4)], and into *L. lactis* Lb790 as described [[5](#_ENREF_5)]. The pSIP411 vector [[6](#_ENREF_6)] contains the high-copy-number lactococcal replicon pSH71 [[7](#_ENREF_7)], which allows replication in both lactobacilli and lactococci. It also contains the P_sppQ_ (previously designated P_orfX_) promoter from the *spp* regulon [[8](#_ENREF_8)], which is activated by the sakacin P-inducing peptide (SppIP) in lactobacilli, but not in lactococci (Axelsson, L., personal communication). Recombinant plasmids were selected on MRS agar that contained 10 µg/ml erythromycin.

The purification of plasmids from lactobacilli and lactococci required an additional lysis step before the lysis buffer provided in the kit was added. These bacteria were incubated at 37°C for 10 min and 25 min, respectively, in buffer (50 mM glucose, 25 mM Tris-HCl [pH 8], 10 mM EDTA) hat contained lysozyme (20 mg/ml), mutanolysin (40 U/ml) and RNase (100 µg/ml), as described previously [[9](#_ENREF_9)]. Recombinant plasmids were checked for an inserted fragment of the correct size using PCR amplification with primers specific for the pSIP411 vector.

To construct a plasmid that produces the 224-bp OVA_f_ in *E. coli*, the pSIP409-p9 vector [[10](#_ENREF_10)] was utilised. It contains a pGEM-series (Promega) replicon, which permits stable high-copy-number replication in *E. coli*, as well as the 256_rep_ [[11](#_ENREF_11)], which permits replication in some lactobacilli, such as *L. plantarum* and *L. sakei*. The pSIP409-p9 vector contains the p9 constitutive promoter [[10](#_ENREF_10)], which supports protein expression in *E. coli*, lactobacilli, and lactococci. The 224-bp OVA_f_ was ligated into the *Nco*I and *Xho*I sites of the pSIP409-p9 vector and transformed into *E. coli* XL10 Gold. Transformants were selected on BHI agar that contained 200 µg/ml erythromycin, and checked for the presence of a fragment of the correct size by PCR amplification using the pSIP409 primers. In addition, the pSIP409-p9-sGFP vector (Axelsson, unpublished), which encodes a synthetic *GFP* gene and is modified for *Lactobacillus* codon usage, was transformed into *E. coli* XL10 Gold.

Although pSIP409-p9 functions in *L. plantarum* and *L. sakei*, it is present in only low copy number and yields moderate levels of protein expression. To construct plasmids with high constitutive production of OVA_f_ and GFP in lactobacilli and lactococci, elements of pSIP411 and pSIP409p9-OVA_f_/GFP were combined. DNA fragments that contained p9-OVA_f_ and p9-GFP were restricted from pSIP409-p9OVA_f_ and pSIP409-p9GFP using the *Bgl*II and *Xho*I enzymes, purified, ligated to *Bgl*II- and *Xho*I-digested pSIP411, and electroporated into *L. plantarum* NC8. Recombinant plasmids were selected on erythromycin-containing agar and analysed by PCR. The resulting pSIP411-p9OVA_f_ and pSIP411-p9GFP plasmids were also electroporated into *L. sakei* Lb790 and *L. lactis* MG1363.

The OVA_f_- and GFP-expressing plasmids were sequenced to ensure that no mutations had occurred. The promoter and OVA_f_/GFP sequences were amplified with appropriate pSIP409 or pSIP411 primers. The amplified fragments were sequenced using the ABI Prism BigDye Terminator Cycle Sequencing Ready Reaction kit (PE Applied Biosystems), following the manufacturer’s instructions. No mutations were detected in any of the constructs.

**Production of His-tagged OVA_319-386_**

The OVA_319-386_ peptide, which used as a molecular mass marker, was produced in the His-tag inducible expression plasmid pQE30 (QIAexpressionist kit; Qiagen). The OVA_f_-fwd1 primer (5´-TAGGGATCCGCTGAATCATTGAAAATCTC-3´; *Bam*HI site underlined) and the nis9r primer (both from MWG-Biotech) were used with the pSIP411-OVA_f_ vector as the template. The fragment was purified in a QiaQuick PCR purification column (Qiagen), digested with *Bam*HI and *Kpn*I, gene-cleaned, ligated into the *Bam*HI-*Kpn*I site of pQE30. and transformed into *E. coli* XL10 Gold. Expression and purification of His-OVA_f_ were performed according to the manufacturer’s instructions.

1. Catterall JF, O'Malley BW, Robertson MA, Staden R, Tanaka Y, et al. (1978) Nucleotide sequence homology at 12 intron--exon junctions in the chick ovalbumin gene. Nature 275: 510-513.

2. Mercereau-Puijalon O, Royal A, Cami B, Garapin A, Krust A, et al. (1978) Synthesis of an ovalbumin-like protein by Escherichia coli K12 harbouring a recombinant plasmid. Nature 275: 505-510.

3. Perez-Arellano I, Zuniga M, Perez-Martinez G (2001) Construction of compatible wide-host-range shuttle vectors for lactic acid bacteria and Escherichia coli. Plasmid 46: 106-116.

4. Aurkust TaH, Blom (1992) Transformation of Lactobacillus strains used in meat and vegetable fermentations. Food Res Int 25: 253-261.

5. Holo H, Nes IF (1989) High-Frequency Transformation, by Electroporation, of Lactococcus lactis subsp. cremoris Grown with Glycine in Osmotically Stabilized Media. Appl Environ Microbiol 55: 3119-3123.

6. Sorvig E, Mathiesen G, Naterstad K, Eijsink VG, Axelsson L (2005) High-level, inducible gene expression in Lactobacillus sakei and Lactobacillus plantarum using versatile expression vectors. Microbiology 151: 2439-2449.

7. de Vos W (1987) Gene cloning and expression in lactic streptococci. FEMS Microbial Rev 46: 281-295.

8. Risoen PA, Brurberg MB, Eijsink VG, Nes IF (2000) Functional analysis of promoters involved in quorum sensing-based regulation of bacteriocin production in Lactobacillus. Mol Microbiol 37: 619-628.

9. Axelsson L, Holck A, Birkeland SE, Aukrust T, Blom H (1993) Cloning and nucleotide sequence of a gene from Lactobacillus sake Lb706 necessary for sakacin A production and immunity. Appl Environ Microbiol 59: 2868-2875.

10. Rud I, Jensen PR, Naterstad K, Axelsson L (2006) A synthetic promoter library for constitutive gene expression in Lactobacillus plantarum. Microbiology 152: 1011-1019.

11. Sorvig E, Skaugen M, Naterstad K, Eijsink VG, Axelsson L (2005) Plasmid p256 from Lactobacillus plantarum represents a new type of replicon in lactic acid bacteria, and contains a toxin-antitoxin-like plasmid maintenance system. Microbiology 151: 421-431.
